# A Novel Toll-Like Receptor (TLR) Influences Compatibility between the Gastropod *Biomphalaria glabrata*, and the Digenean Trematode *Schistosoma mansoni*

**DOI:** 10.1371/journal.ppat.1005513

**Published:** 2016-03-25

**Authors:** Emmanuel A. Pila, Mahmoud Tarrabain, Alethe L. Kabore, Patrick C. Hanington

**Affiliations:** The School of Public Health, University of Alberta, Edmonton, Alberta, Canada; George Washington University School of Medicine and Health Sciences, UNITED STATES

## Abstract

Schistosomiasis, a devastating disease caused by parasitic flatworms of the genus *Schistosoma*, affects over 260 million people worldwide especially in tropical and sub-tropical regions. Schistosomes must undergo their larval development within specific species of snail intermediate hosts, a trait that is shared among almost all digenean trematodes. This unique and long-standing host-parasite relationship presents an opportunity to study both the importance of conserved immunological features in novel immunological roles, as well as new immunological adaptations that have arisen to combat a very specific type of immunological challenge. While it is well supported that the snail immune response is important for protecting against schistosome infection, very few specific snail immune factors have been identified and even fewer have been functionally characterized. Here, we provide the first functional report of a snail Toll-like receptor, which we demonstrate as playing an important role in the cellular immune response of the snail *Biomphalaria glabrata* following challenge with *Schistosoma mansoni*. This TLR (BgTLR) was identified as part of a peptide screen of snail immune cell surface proteins that differed in abundance between *B*. *glabrata* snails that differ in their compatibility phenotype to challenge by *S*. *mansoni*. The *S*. *mansoni*-resistant strain of *B*. *glabrata* (BS-90) displayed higher levels of BgTLR compared to the susceptible (M-line) strain. Transcript expression of BgTLR was found to be very responsive in BS-90 snails when challenged with *S*. *mansoni*, increasing 27 fold relative to β-actin (non-immune control gene); whereas expression in susceptible M-line snails was not significantly increased. Knockdown of BgTLR in BS-90 snails via targeted siRNA oligonucleotides was confirmed using a specific anti-BgTLR antibody and resulted in a significant alteration of the resistant phenotype, yielding patent infections in 43% of the normally resistant snails, which shed *S*. *mansoni* cercariae 1-week before the susceptible controls. Our results represent the first functional characterization of a gastropod TLR, and demonstrate that BgTLR is an important snail immune receptor that is capable of influencing infection outcome following *S*. *mansoni* challenge.

## Author Summary

The freshwater snail *Biomphalaria glabrata* is the subject of intensive research, primarily due to its biomedical importance as an intermediate host for the parasitic flatworm *Schistosoma mansoni*, which is a causative agent of the disease schistosomiasis–a chronic, debilitating condition that affects over 260 million people worldwide. Studies of this snail have led to the identification of many factors, some with high homology to known immune molecules in other organisms and many more that are unique with no known homology. However, research into the functional and mechanistic roles of these factors has only recently progressed. In this study, we have demonstrated the functional relevance of a Toll-like receptor (TLR) that we identified in *B*. *glabrata*. Transcriptional expression of this TLR was rapidly induced in a snail strain that is resistant to *S*. *mansoni*, in a pattern that is consistent with their phenotype. Furthermore, knockdown of the TLR resulted in 43% of resistant snails becoming infected with *S*. *mansoni* to patency. This advances our understanding of the mechanistic basis of snail-schistosome compatibility significantly, and may one day facilitate the development of tools for improving the control of schistosomiasis. It also contributes novel functionality to TLRs, one of the most evolutionarily conserved pattern-recognition receptors and cognate signalling pathways in immunity.

## Introduction

Schistosomiasis is a devastating disease caused by parasitic flatworms of the genus *Schistosoma*. It affects over 260 million people worldwide especially in tropical and sub-tropical regions [1,2]. The freshwater snail *Biomphalaria glabrata* acts an obligate intermediate host for *Schistosoma mansoni*, which is the causative agent of intestinal schistosomiasis in Africa and South America. This association to human schistosomiasis has made *B*. *glabrata* one of the most extensively studied gastropods in terms of immunobiology and host-parasite interactions. This snail continues to play an important role as a model for studying the intra-molluscan aspects of the parasite lifecycle, which has gained popularity as a possible target for disease control purposes [3]. Strains of *B*. *glabrata* have been bred that display differing compatibility phenotypes to *S*. *mansoni* infection. Strains displaying resistance such as BS-90, 13-16-R1, 10-R2 [4,5] or susceptibility such as the M-line and NMRI [6,7], serve as a means to evaluate and better understand the driving mechanisms underpinning snail resistance and susceptibility. They provide valuable tools for elucidating the specifics of these naturally occurring processes [8].

An improved understanding of the molecular basis for susceptibility/resistance is considered important due to the potential for the development of novel control strategies for schistosomiasis. Moreover, such knowledge would contribute significantly to the field of evolutionary and invertebrate immunology, particularly of the Lophotrochozoa, a superphylum of Metazoa to which molluscs belong. Compared to the other two superphyla [Ecdysozoa (represented by fruit flies and nematodes) and Deuterostoma (represented by vertebrates)], with respect to our understanding of immunological capability and function, this group remains under-represented in the literature [9].

Studies utilizing the *B*. *glabrata*/*S*. *mansoni* and other *Biomphalaria*/trematode models have made significant progress in the past two decades, with the focus shifting from classic comparative immunology to one with molecular insight [8,10]. These advances have been made possible by the availability and integration of techniques such as proteomics [11–14], functional genomics [15–19] and population genetics [20–24]. Gene discovery, comparative proteomics and differential gene expression studies with these snails, their haemocytes, or various tissues, have led to the identification of hundreds of immune factors with putative influence on snail-schistosome compatibility [12,14,16, 25–30].

Few studies have dealt with the functional and mechanistic roles of these factors using defined bioassays. Of those that have been characterized, the fibrinogen-related proteins (FREPs) are perhaps the best understood. FREPs comprise a diverse family of secreted lectins that have been shown to be important in the anti-trematode response [31,32]. They are known to act as opsonins, recognizing schistosome surface proteins [31] and excretory/secretory products [33], forming complexes with another diverse family of proteins on larval schistosomes–the *Schistosoma mansoni* polymorphic mucins [34]. *Biomphalaria glabrata* migration inhibitory factor (BgMIF) is another factor for which functional involvement in the anti-schistosome response has been demonstrated. BgMIF was shown to stimulate cellular proliferation by activating extracellular signal-regulated kinase and to suppress nitric oxide-induced apoptosis in *Biomphalaria glabrata* embryonic cells. Knockdown of BgMIF reduced encapsulation of sporocysts by these cells and increased parasite burden in the snails [35]. Other molecules that have been studied functionally include the copper/zinc superoxide dismutase and its products [36–38], and the beta pore-forming toxin biomphalysin [39]. To date, a mechanism or receptor demonstrating how these factors might engage with and elicit the cellular encapsulation response that appears to be necessary for clearance of an invading trematode has not been identified.

Transition into studies of this nature is required in order to piece together the big picture of how the snail immune response works, and to fully understand the molecular basis of snail-trematode interactions. The evidence thus far indicates that a number of conserved pathways involved in the vertebrate immune response or their interacting components are also present in *B*. *glabrata*. These include pathways of the reactive oxygen and nitrogen intermediates mediated by superoxide dismutase, and macrophage migration inhibitory factor, mentioned above. The mitogen-activated protein kinase pathway is also conserved in the snail and shown to be relevant in haemocyte motility, spreading, phagocytosis, and encapsulation [40–43].

One of the major evolutionarily conserved pathways in innate immunity is the Toll/Toll-like receptor (TLR) pathway. TLRs are trans-membrane proteins composed of an extracellular leucine-rich repeat domain, responsible for pathogen recognition and a conserved cytoplasmic Toll/IL-1 (TIR) domain, which is responsible for signal transduction and activation of effector functions. In 2007, Zhang and colleagues [44] published homologues of Gram-negative bacteria binding protein and peptidoglycan recognition protein in *B*. *glabrata*. Both are extracellular components that can activate TLR and the related immune-deficiency (IMD) pathways respectively. Later, they also identified the nuclear factor kappa B (NF-kB), a major downstream transcription factor in the TLR pathway. NF-kB expression patterns in resistant snails infected with schistosome parasites was found to be consistent with the early immune response patterns critical for parasite killing [9]. However, it was not known whether, and how, TLR was involved in regulating parasite infection in this snail. While comparing haemocyte surface proteins between *S*. *mansoni*-susceptible and resistant *B*. *glabrata* snails, we discovered peptides of a TLR-like protein, some of which displayed a high amino acid sequence identity with the TIR domain of known vertebrate TLRs. Peptides associated with this protein were at a significantly higher abundance in the resistant snail strain, prompting us to further investigate this putative TLR.

In this study, we have conducted the first functional characterization of a TLR in the snail *B*. *glabrata*. TLRs play a key role in the innate immune response by directly recognizing a variety of pathogens (typically bacteria, viruses and fungi) or factors evidencing their presence and transducing signals to the immune cells [45]. The leucine-rich repeat (LRR) region of TLRs recognizes conserved motifs on pathogens known as the pathogen-associated molecular patterns (or PAMPs) such as bacterial lipopolysaccharide, flagellin protein and genomic DNA containing unmethylated CpG motifs [46–48], viral DNA [49], fungal zymosan [50] and the plasmodium pigment hemozoin [51]. The Toll/TLR pathway can also be activated by host endogenous ligands or molecules that signal tissue damage–the so-called damage-associated molecular patterns (DAMPs). These include molecules such as uric acid, fibrinogen, mitochondrial DNA, heat-shock proteins 60 and 70 and fibronectin [52]. *Drosophila* Toll, the founding member of the TLR family is not a direct recognition receptor. Instead, pathogen components trigger the activation of protease cascades that then lead to the cleavage of the Toll ligand–Spaetzle [53]. The cytoplasmic region of TLRs which shares homology with the interleukin-1 receptor (known as a Toll/interleukin-1 receptor (TIR) domain) [45] is responsible for transducing signals from pathogen recognition to the immune cells leading to the activation of the effector functions. This pathway is evolutionarily conserved from nematodes to mammals [54].

The *B*. *glabrata* TLR we have characterized in this study (GenBank accession number: JX014259.1, herein after referred to as BgTLR) possesses complete LRR and TIR domains, and we demonstrate here the involvement of BgTLR in the immune response of *B*. *glabrata* against *S*. *mansoni*. This implies that BgTLR has a novel functionality with respect to helminth parasites, making it unique among known TLRs, one of the most evolutionarily conserved pattern-recognition receptors and cognate signalling pathways in immunity.

## Results

### BgTLR was identified as part of a haemocyte protein expression screen comparing resistant and susceptible snails

Membrane-associated proteins displaying differential expression in haemocytes of BS-90 *B*. *glabrata* when compared to the M-line strain were identified using iTRAQ and LC/MS/MS. Including BgTLR, 16 proteins were identified with confidence, as being present at higher abundance in BS-90 snail haemocytes (S1 Table). Four proteins could not be matched with any factors that have a known function, however, of the 12 identified proteins superoxide dismutase Cu/Zn [36, 55, 56], dermatopontin 2, matrilin, an elastase-like protein [30, 31], and a protein with significant similarity to protein tyrosine phosphatase domain-containing proteins [57], have all been associated with resistant snail phenotypes in other studies.

### BgTLR displayed increased transcript expression in BS-90 snails following challenge with *S*. *mansoni*

**Fig 1:**
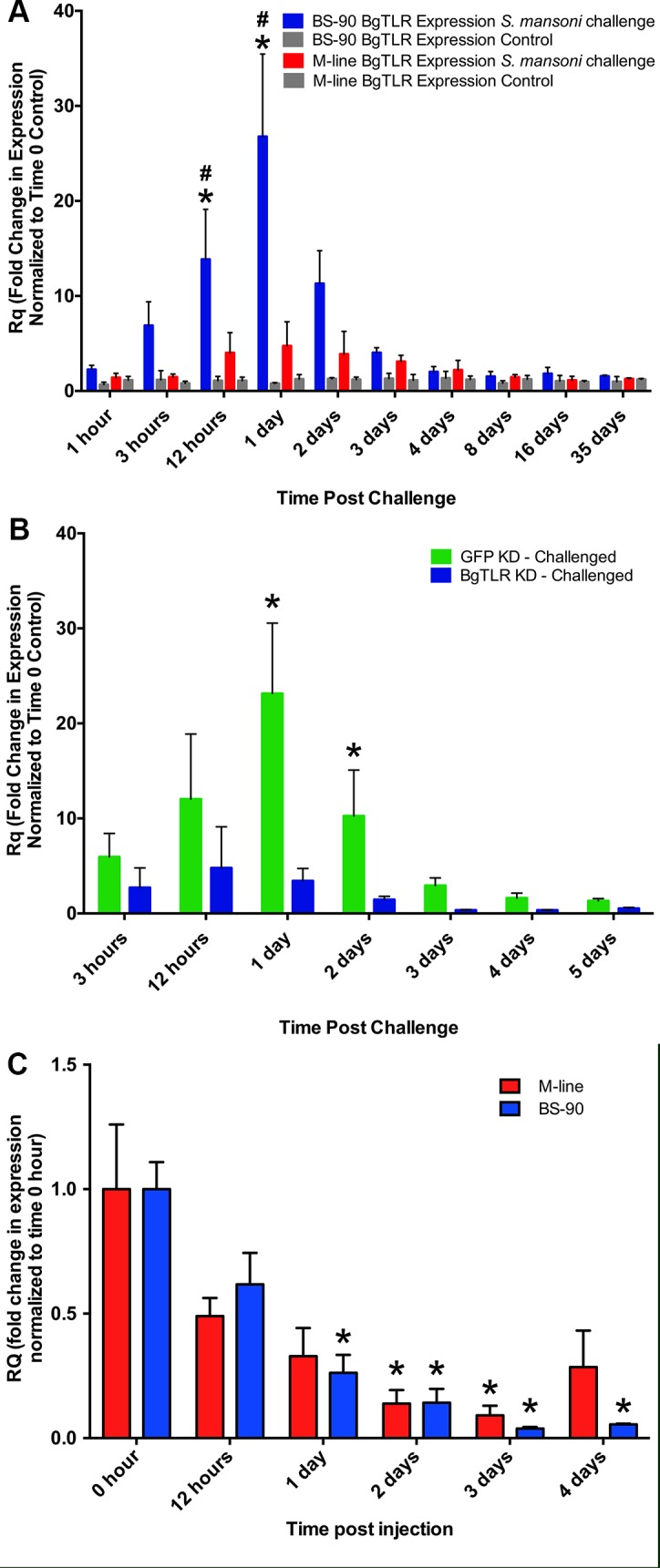
BgTLR displayed increased transcript expression in BS-90 snails following challenge with *S*. *mansoni*. (A) BgTLR transcript expression with and without *S*. *mansoni* challenge. Snails (BS-90 and M-line strains) were individually exposed to ~5 miracidia or left unexposed (control). Five snails were collected at indicated time points over the incubation period of the parasite. RNA was extracted from whole snails, converted to cDNA and BgTLR expression was measured by quantitative PCR. Expression was quantified in fold changes normalized to time 0-hour controls. Bars represent standard error (n = 5). Asterisk (*) indicates significant difference (P < 0.05) between experimental and control samples, while hash (#) indicates significant difference between BS-90 and M-line snails at the respective time points. (B) Knockdown of BgTLR or GFP (control) in BS-90 snails responding to *S*. *mansoni* parasite challenge. (C) Knockdown of BgTLR in unexposed M-line and BS-90 snails. Five snails were collected for each time point in B and C for RNA extraction and cDNA synthesis. Bars represent standard error (n = 5). Asterisks indicate significant difference (P < 0.05) from 0-hour time points.

To determine whether BgTLR transcript expression was responsive to *S*. *mansoni* challenge, we used quantitative polymerase chain reaction (qPCR) to measure its expression patterns in schistosome-challenged snails, which were compared to control non-challenged snails at each experimental time point, and the endogenous control β-actin. BgTLR expression was rapidly induced in the *S*. *mansoni*-resistant (BS-90) snails responding to *S*. *mansoni* challenge, with transcript abundance significantly increasing 14 fold as early as 12 hours post challenge compared to the 0-hour time point. The highest value recorded was a 27 fold increase at 1 day post challenge, which was significantly different from all other time points (P < 0.05). This was followed by reduced BgTLR transcript abundance that returned to pre-exposure levels by day 8 post challenge (dpc) (Fig 1A). In contrast, *S*. *mansoni* challenge did not induce significant changes in BgTLR transcript expression in the susceptible (M-line) snails (Fig 1A). Non-exposed BS-90 and M-line snails expressed very similar levels of BgTLR transcript as determined by comparison of absolute expression (C_t_ values) which ranged approximately 7.2 to 7.5.

### Predicted structure of BgTLR

The full-length coding sequence of BgTLR is composed of 3546 nucleotides. The open reading frame predicts a protein of 1181 amino acid residues. Its ectodomain contains 23 LRR motifs spanning residues 3–725 as predicted by LRRfinder [58] and ScanProsite tools [59]. The TIR domain is encoded by residues 899–1037, preceded by a transmembrane region on residues 849–870 (S1 Fig). There is no identifiable signal peptide in BgTLR based on current predictive software. BlastP analyses [60] indicate that the TIR region of BgTLR shares the highest identity to other molluscan TLR TIR regions (65–88%), followed by those of arthropods (51–58%). Among the mammals, TLRs having closest TIR region identities to BgTLR are TLR 4 (40%), TLR 3 (38%) and TLR 13 (37%). The LRR region of BgTLR is much more variable, with identity range of 27–43% only to other molluscan and arthropod TLRs.

Amplification specificity was confirmed using qPCR melt curve and customized BLAST sequence analyses (S2 Fig and S3 Fig) while siRNA knockdown specificity was assessed by measuring the abundance of the three sequences in the *B*. *glabrata* genome that shared the highest nucleotide identity with BgTLR using BgTLR knockdown cDNA as a template (S5 Fig). The peptide fragment used for the synthesis of the antibody used in this study for Western blot and immunocytochemistry analyses was also confirmed to be specific by testing the binding of peptide pre-incubated antibody to BgTLR (S7 Fig).

### siRNA-mediated knockdown of BgTLR

**Fig 2:**
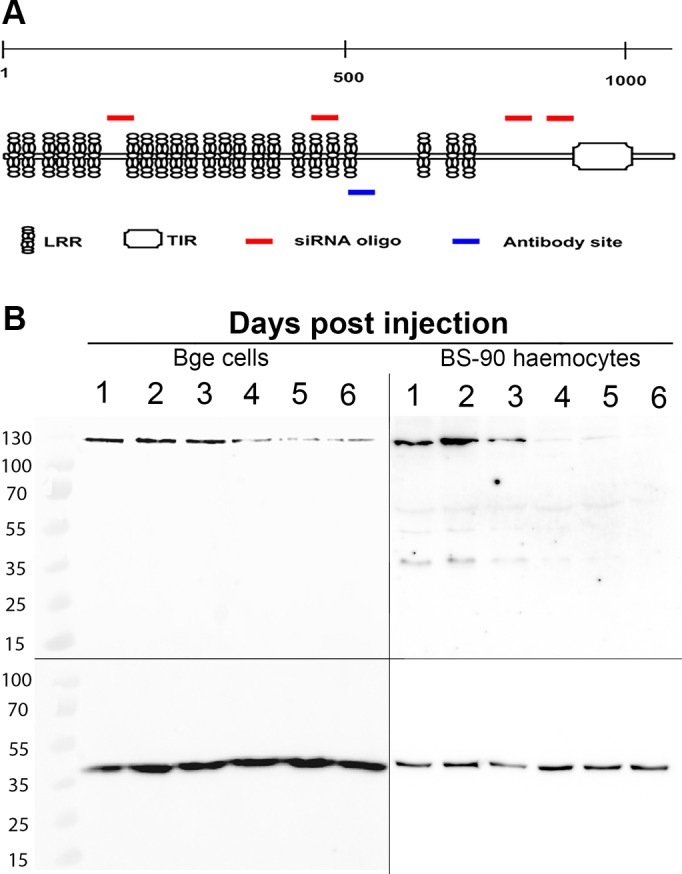
siRNA-mediated knockdown of BgTLR transcripts. (A) Graphic view of BgTLR (not drawn to scale) showing the leucine-repeat motifs and Toll/IL-1 domain. Red bars indicate the approximate positions targeted by siRNA oligonucleotides used to inject the snails, while the blue bar indicates the targeted position of the antibody used. (B) Confirmation of BgTLR (~135 kDa) knockdown in BS-90 haemocytes and Bge cells respectively. Protein was detected via Western blot with primary antibody developed against the extracellular region of BgTLR. BgActin (~42 kDa) served as the loading control.

Injection of an siRNA oligonucleotide cocktail targeting the extracellular LRR region of BgTLR (Fig 2A) into M-line and BS-90 snails induced a measurable knockdown of BgTLR expression in both snail strains. Maximum knockdown was observed at 3 days post injection with transcript levels of 0.09 and 0.04 fold compared to the time 0-hour control for M-line and BS-90 strains respectively. Both were significantly different from time 0-hour control (P < 0.05) (Fig 1C). Knockdown effect of BgTLR siRNA was sustained in BS-90 snails following *S*. *mansoni* challenge with BgTLR transcript levels reducing down to 0.4 fold at 3-days post injection. In comparison, snails injected with siRNA oligonucleotides targeted to GFP as a control displayed a 3 fold BgTLR expression increase at 3 days post injection (Fig 1B). BgTLR knockdown kinetics at the protein level in BS-90 snails lagged behind transcript knockdown with an observable reduction in BgTLR protein abundance at 4 days post injection as determined by Western blots using both haemocytes isolated from BS-90 snails and using *B*. *glabrata* embryonic (Bge) cell line (Fig 2B).

### BgTLR immunolocalization in haemocytes and Bge cells

**Fig 3:**
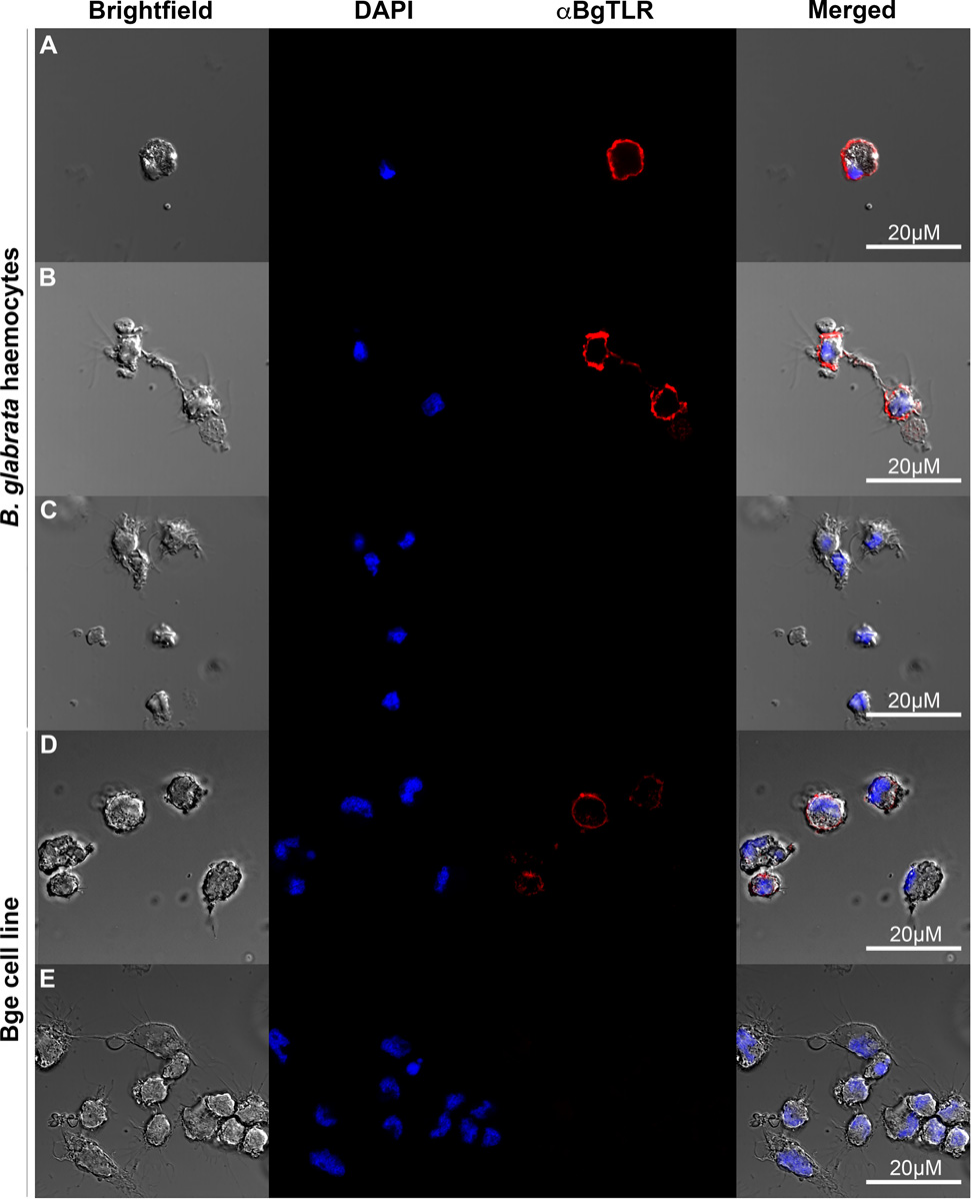
BgTLR immunolocalization in haemocytes and Bge cells. Haemocytes (A-C) and Bge cells (D-E) were labelled with DAPI and anti-BgTLR primary antibody. Control haemocyte (C) and Bge (E) samples were stained with DAPI but the primary antibody step omitted. Scale bars represent 20 μM.

Immunocytochemical studies using an antibody developed against the extracellular region of BgTLR (Fig 2A) indicate that it is expressed in the majority of haemocytes found in the circulation of *B*. *glabrata*, although some haemocytes appeared not to express BgTLR (S6 Fig). In the Bge cell line, all cells appear to be BgTLR-positive with differences in staining intensity among cells (Fig 3). BgTLR positive signals were detected primarily on the extracellular surface of the plasma membrane, and faint immunolocalization was observed in the cytoplasm of both haemocytes and Bge cells (Fig 3 and S1 Video). Intracellular localization may be due to BgTLR trafficking between the cellular membrane and intracellular compartments similar to TLR 4 expression in human monocytes [61] or it may indicate that BgTLR has a role in the intracellular environment. We currently have studies underway focusing on identifying the BgTLR ligand(s) which might clarify its subcellular localization and possible intracellular role.

### Knockdown of BgTLR in BS-90 snails decreases haemocyte phagocytic response

**Fig 4:**
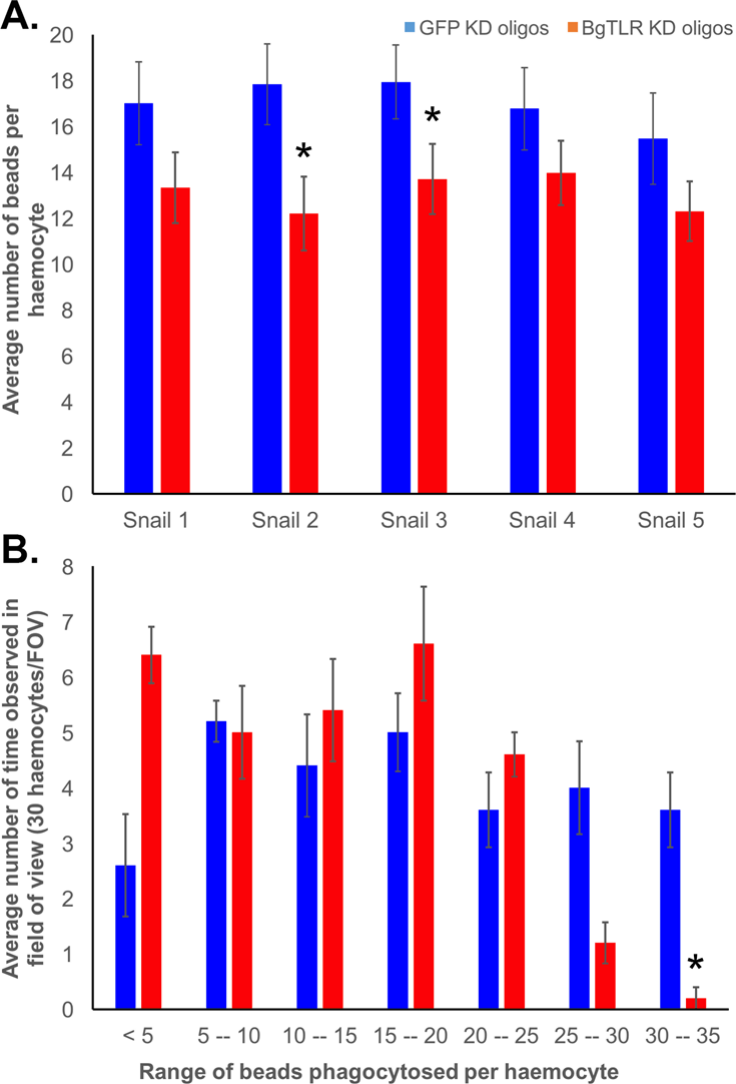
Knockdown of BgTLR in BS-90 snails decreases haemocyte phagocytic response. (A) Mean number of phagocytosed beads per haemocyte. Five BS-90 snails each were injected with siRNA targeting BgTLR or GFP (control) and 96 hours later, haemolymph was extracted from the snails and immediately mixed with ~1 x 10^6^ 1μM FITC-labelled streptavidin-coated beads pre-incubated with biotinylated *S*. *mansoni* excretory/secretory products and sporocyst. After 3 hours, haemocytes from each snail were counted from a random field of view on the slide, and 30 haemocytes for each snail were assessed for the number of beads within each cell from which mean number of beads per haemocyte was calculated. Asterisk (*) indicates significant reduction (P < 0.05) in the mean number of beads per haemocyte in BgTLR knockdown snails. (B) Frequency of number of beads observed in 30 haemocytes from one field of view (FOV). Asterisk (*) indicates significant difference (P < 0.05) in the average number of beads phagocytosed between BgTLR and GFP knockdown.

Knockdown of BgTLR significantly (P<0.05) decreased the mean number of *S*. *mansoni* sporocyst excretory/secretory/lysate-coated beads phagocytosed by individual haemocytes of BS-90 snails. Snails injected with siRNA oligonucleotides targeting BgTLR and 96 hours later assessed for phagocytic activity averaged 13.1 beads per haemocyte, whereas, GFP knockdown control snails averaged 17 beads per haemocyte (Fig 4A). A breakdown of the number of beads observed in each haemocyte demonstrated that haemocytes from BgTLR KD snails were much less likely to take in more than 25 beads per haemocyte compared to the GFP controls (Fig 4B).

### Knockdown of BgTLR in resistant BS-90 snails yields cercariae-producing *S*. *mansoni* infections

**Fig 5:**
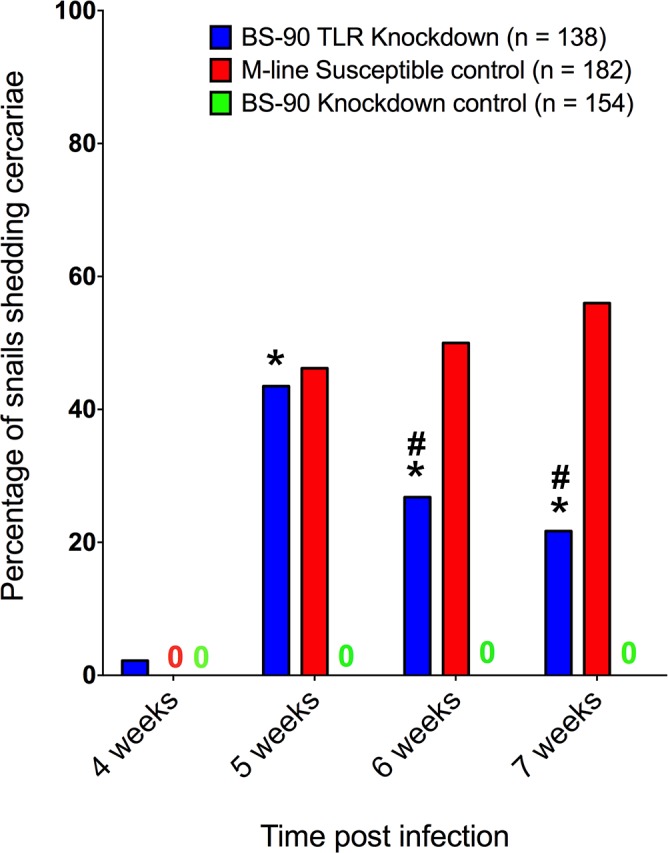
Knockdown of BgTLR in resistant BS-90 snails yields cercariae-producing *S*. *mansoni* infections. BS-90 snails were injected with BgTLR or GFP (knockdown control) siRNA oligonucleotide mix and after 48 hours exposed individually to ~5 miracidia. Non-injected M-line snails served as the susceptible controls for parasite viability. The number of cercariae-producing snails was expressed as a percentage of the starting number (n) of snails. Figure represents pooled data from two independent experiments. Asterisk (*) indicates significant difference from BS-90 GFP knockdown control for each week while hash (#) indicates significant difference between BS-90 BgTLR knockdown and M-line susceptible control for each week.

Knockdown of BgTLR abrogated the resistant phenotype of BS-90 snails to *S*. *mansoni* infection. Snails injected with siRNA oligonucleotides and challenged with *S*. *mansoni* 2-days later developed patent infections that led to cercariae shedding in ~ 43% of BS-90 snails over two trials, whereas none of the control GFP-siRNA injected BS-90 snails shed cercariae (Fig 5). Knockdown of BgTLR resulted in cercariae shedding in the BS-90 snails one week prior to the susceptible M-line snails, which served as a benchmark for infection success. At week 4 post exposure, 2% of the BS-90 BgTLR knockdown snails shed cercariae. This increased to 43% at week 5 and then decreased to 22% at week 7 when the experiment was terminated. The proportions of BS-90 BgTLR knockdown snails shedding cercariae at weeks 5, 6 and 7 were statistically significant (P<0.05) compared to BS-90 GFP knockdown snails. In comparison, M-line snails started shedding cercariae only at week 5 (46%), increasing to 56% at week 7 (Fig 5). There was no statistically significant difference at week 5 in the proportions of snails shedding cercariae between BS-90 BgTLR knockdown and the M-line susceptible control. Injection of siRNA oligonucleotides resulted in an overall mortality of 27%, 17% and 19% for BS-90 BgTLR knockdown, BS-90 GFP knockdown and the M-line susceptible controls respectively. It is worth noting that these mortalities might have impacted the percentages of shedding snails which were calculated out of the total starting number. Since snails were not tracked individually over the course of the experiment, these percentages likely underestimate shedding snails that were lost in the mortalities.

## Discussion

*Biomphalaria glabrata* has received tremendous research attention due to its biomedical importance as an intermediate host of the causative agent of human schistosomiasis [1,2]. In the wake of the World Health Organization’s call for global elimination of schistosomiasis by the year 2025 [62], there is intensified interest to understand the molecular basis of the interactions that take place between schistosomes, and their snail hosts, with a view to identifying targets or mechanisms that could be exploited in disease prevention and control efforts. As a primary determinant of compatibility between this host and parasite, the snail internal defense response constitutes one area worthy of in-depth investigation in this context.

In this study, we have characterized a TLR in *B*. *glabrata* that we demonstrate is immunologically relevant during the anti-schistosome immune response of the snail. Our study provides significant evidence that BgTLR is involved in regulating parasite infection in this snail. BgTLR expression was increased as early as 3 hours following *S*. *mansoni* challenge in the resistant (BS-90) snails, lasting up to 3 days before returning to pre-exposure levels (Fig 1A). This pattern of expression is consistent with the involvement of BgTLR in the immune response because the duration coincides with the critical period for the clearance of parasite infection in incompatible strains [63–65]. Moreover, the means by which BgTLR was initially identified suggested that it was present in higher abundance in BS-90 snails when compared to M-line. This is further supported by siRNA-mediated knockdown of BgTLR, which also had maximal effect within this duration (Fig 1B and 1C). Forty-three percent of resistant snails that received BgTLR-specific siRNA oligonucleotides before *S*. *mansoni* challenge went on to shed cercariae after 4 weeks incubation period. On the other hand, resistant snails that did not receive BgTLR-specific siRNA but challenged with *S*. *mansoni* cleared the infection within 4 days as determined by a qPCR assay targeting the parasite glyceraldehyde 3-phosphate dehydrogenase (GAPDH) gene which failed to detect parasite product beyond day 4 (S4 Fig). Conversely, in susceptible (M-line) snails, GAPDH expression was still detectable after 4 days post-infection and increased throughout the incubation period.

The siRNA oligonucleotides used to knockdown BgTLR transcript are specific as shown with qPCR amplification (S5 Fig) of the three predicted transcripts sharing the highest nucleotide identity with BgTLR available on the Vectorbase database for *B*. *glabrata* (www.vectorbase.org). However, their cross-reaction with BGLB008602 leaves the possibility that the phenotypic knockdown effects observed may be due to either or both of these proteins. BGLB008602 is 100% identical to BgTLR but lacks a 1195 bp region of BgTLR and cannot be functionally distinguished from BgTLR using our siRNA knockdown approach. We have reason to believe that both may be splice variants of the same gene based on our finding that the ‘missing’ 1195 bp region is currently annotated as introns 8 and 9 in the *B*. *glabrata* genome (www.vectorbase.org). As shown by Western blot (Fig 2 and S7 Fig), no band representative of the size predicted for BGLB008602 (~ 127 kDa) is observed, suggesting that its effect if any is minimal, or in a different context than the anti-*S*. *mansoni* immune response. Should these transcripts be true splice variants, the difference in size would most likely impact their interaction with pathogen-associated molecular patterns (PAMPs) or endogenous ligands since the differential region is in the extracellular domain and contains leucine-rich motifs commonly required for ligand recognition.

Currently, we do not know the precise recognition mechanism by which BgTLR is involved in the snail immune response. That is, what PAMPs or perhaps endogenous ligands it recognizes and how this signal is transduced to the haemocyte. TLRs have been characterized in a number of other molluscs such as the disk abalone, Hawaiian bobtail squid, Zhikong scallop, soft-shell clam, mussel and oyster [66–70], however this is the first functional identification of a TLR in a gastropod. To date, all molluscan TLRs that have been functionally assessed are thought to be involved in antimicrobial immune responses. This is typical of most TLRs as they recognize PAMPs ranging from lipids, proteins, lipoproteins and nucleic acids [54]. An interesting feature of BgTLR is that it is induced in response to *S*. *mansoni* challenge. This suggests that it might be involved in recognizing PAMPs that are shared between schistosomes and microbes. However, an intriguing possibility is that BgTLR may be exclusive for schistosome (or digenean trematode)-specific epitopes. Should this be the case, it will mark a significant finding in the field of invertebrate immunology, and add a new PAMP to the TLR ligand cannon. To the best of our knowledge, no anti-parasitic recognition/response involving TLRs have been characterized in an invertebrate.

There are two possible sources of exogenous ligand(s) that might form the basis of TLR-based recognition in *B*. *glabrata* with respect to schistosomes. The first includes the complex carbohydrates which are major components of larval teguments [71–73]. Secondly, during larval transformation, complex glycoproteins are also released with the other larval transformation products [74] which might be recognized by BgTLR. Larval carbohydrates have been shown to elicit functional immune responses such as the induction of reactive oxygen species production in haemocytes [75–78] and phagocytosis [79]. Both parasite larval carbohydrates and glycoproteins can bind to snail plasma and haemocyte proteins *in vitro* [80–83].

It also remains possible that the BgTLR ligand(s) might be endogenous. This is also the case for the founding member of the TLR family (*Drosophila* Toll) and for some mammalian TLRs. In *Drosophila*, the Toll does not function as a direct recognition receptor of a microbial pattern. Instead, its ligand (spaetzle) is activated first through proteolytic cleavage by pathogen-triggered protease cascades [53]. Examples of molecules that serve as endogenous ligands abound in mammals. These include uric acid, mitochondrial DNA, heat-shock proteins 60 and 70, fibronectin and fibrinogen [52]. In mice, evidence suggests that TLR4 recognizes fibrinogen to induce chemokine secretion in macrophages [84]. Based on this evidence, one candidate group of molecules that may serve as an endogenous ligand for BgTLR is the fibrinogen-related proteins, which are highly diversified family of lectins functionally linked to the anti-parasite response in *B*. *glabrata* [31,32]. We are currently undertaking studies with the objective to screen for and identify BgTLR ligand(s).

In terms of functional immune responses driven by BgTLR, our experimental results support the involvement of phagocytosis as a downstream functional response elicited following engagement, and ultimately resulting in the prevention of parasite infection. Knockdown of BgTLR in BS-90 snails significantly reduced the mean number of beads phagocytosed per haemocyte from 17 to 13 (Fig 4A), although the overall percentage of phagocytic haemocytes did not differ from GFP knockdown control snails. This, and the fact that parasites are too large to be phagocytosed by a single haemocyte indicate that other effector responses might also be activated by BgTLR. Phagocytosis together with cytotoxicity and encapsulation are the major haemocyte responses that are induced as a result of receptor recognition of PAMPS in the snail [85]. It is likely that the role of BgTLR in the snail immune response against *S*. *mansoni* is through the combined effects of these processes. However, the involvement of encapsulation and cytotoxicity remains to be investigated in the context of BgTLR.

The major pathways that are known to be activated downstream of TLR are present in *B*. *glabrata*, and for some, their active involvement in the snail immune response have been demonstrated. These major pathways include the NF-kB, MAPK/ERK and phosphatidylinositol-3 kinase (PI3K). MAPK, ERK and PI3K pathways have been shown to be involved in cellular adhesion, motility and spreading required for phagocytosis and encapsulation [86] as well as in regulating the release of hydrogen peroxide and nitric oxide molecules used in cytotoxicity [77,78]. No functional role has been demonstrated for NF-kB although its expression is consistent with involvement in an immune response [9]. BgTLR may be associated with any of these pathways or their combination in order to activate the effector responses in haemocytes, and this is an area actively under investigation.

BgTLR appears to be selectively expressed in certain haemocytes only (S6 Fig), and differentially on cells of the Bge cell line. The relationship between BgTLR-negative haemocytes with the other haemocyte subsets has not yet been explored. However, expression of pattern recognition receptors on haemocyte subsets is not unique to BgTLR. A member of the fibrinogen-related proteins (FREP3) has been shown to be expressed only by subsets of haemocytes which increase in number during an immune response in the snail [31]. The fact that these immunologically relevant factors are not equally present on all haemocytes of known morphological subset alludes to the likelihood that there is far greater functional diversity within a haemocyte morphotype than is currently understood.

In conclusion, our studies presented here have demonstrated a connection between BgTLR and the resistance phenotype of *B*. *glabrata* with respect to *S*. *mansoni* challenge. By characterizing the receptor which is a major component of the TLR pathway, our findings complement others that have characterized both upstream and downstream components of the pathway [9,77,78,86] and provides conclusive evidence that this pathway is fully conserved and functionally relevant in the immune response of *B*. *glabrata*. It also paves the way for further studies not only to identify the ligands, specific immune responses induced and the haemocyte subsets involved, but also to determine the role of the other *B*. *glabrata* TLRs and leucine rich repeat containing molecules.

## Materials and Methods

### Snails and parasite

Two strains of *Biomphalaria glabrata* snails were used in this study. The BS-90 strain is resistant to *Schistosoma mansoni* infection [4,5] while the M-line strain is susceptible [6,7]. Snails were maintained in aerated artificial spring water at 23–25°C, 12-hour day/night cycle and fed red-leaf lettuce as needed. All snail exposures were performed with the NMRI strain [6] of *S*. *mansoni*, which was maintained at the University of Alberta, cycling between mice and M-line *B*. *glabrata*.

### Identification of BgTLR

BgTLR was identified via a peptide screen of *B*. *glabrata* haemocyte surface proteins using liquid chromatography coupled with tandem mass spectrometry (LC-MS/MS analysis) conducted at the University of Victoria, Canada. Haemocytes were isolated from 10 M-line and 10 BS-90 strain *B*. *glabrata* snails by the head-foot retraction method and immediately mixed 1:1 with chilled 2x Sterile Snail Saline (SSS) and placed on ice. The cells were then centrifuged for 10 minutes at 10,000 x g. The supernatants were aspirated and the haemocytes were resuspended in 0.5 mL 1x SSS. The haemocytes were then sonicated 5 times using 30-second pulses and the resulting homogenate was centrifuged for 30 minutes at 4°C and 10,000 x g. The supernatant was collected and ultracentrifuged at 100,000 x g for 1 hour at 4°C to purify cell membrane. The pellets were resuspended in a buffer containing 8 M urea, 30 mM HEPES, 0.5% SDS, 1 mM PMSF, 2 mM EDTA and 10 mM DTT. This solution was sonicated for 3 minutes using 5-second pulses, interspersed with 3-second breaks. Protein concentration was assessed using a Qubit (Life Technologies) following the manufacturers protocols.

Equal amounts of isolated haemocyte membrane proteins were digested and labelled following the iTRAQ manufacturer’s protocols (Applied Biosystems). 100 μg solutions of each haemocyte population were precipitated using chilled acetone and stored at -20°C for 3 hours. The samples were then centrifuged at 20,000 x g for 45 minutes before resuspension in 20 μL of supplied dissolution buffer. Samples were then processed using the supplied reducing/denaturing kits and cysteines were blocked prior to digestion of the membrane proteins using 1μg/μL sequencing grade trypsin (Promega) overnight at 37°C. Digested protein samples were then labelled with iTRAQ reagents 114 (M-line haemocytes) and 117 (BS-90 haemocytes) following the manufacturers protocols. Upon completion of the labeling procedure, the samples were combined and sent for LC-MS/MS analysis. Peptides identified during analysis were initially compared to a custom Mascot database comprised of known proteins and predicted transcript translations from *B*. *glabrata*, *Lottia gigantica*, and *Aplysia californica*. If no matches were found using this approach, peptide fragments were used in a BlastP search of the GenBank database in order to find the match with the highest amino acid identity. Differential abundance of the membrane proteins between haemocytes of M-line or BS-90 snails was measured as a ratio of the 114:117 labels associated with each analyzed spectra peak.

Peptides relevant to BgTLR, particularly the TIR domain, were used to design primers for rapid amplification of cDNA ends (RACE) PCR (Table 1). RACE successfully amplified the complete BgTLR transcript, which was sequenced and confirmed to be expressed by *B*. *glabrata* using RT-PCR.

**Table 1:** Primers used in this study.

Description	Primer sequence
BgTLR qPCR	Fwd: 5ʹ-GTCTGTCAGGTCGTTGTTCTTA-3ʹ
	Rev: 5ʹ-GATAGACCCTCAAGCTCTGTTG-3ʹ
BgActin qPCR	Fwd: 5'-GCT TCC ACC TCT TCA TCT CTT G-3'
	Rev: 5'-GAA CGT AGC TTC TGG ACA TCT G-3’
Sm GAPDH	Fwd: 5'-TCG TTG AGT CTA CTG GAG TCT TTA CG-3'
	Rev: 5'-AAT ATG ATC CTG AGC TTT ATC AAT GG-3’
BGLB008602 qPCR	Fwd: 5'-GCA GTC GTA AAA GTT GTA GCA G-3'
	Rev: 5'-CCA TGA CCA AAG GAT TTT CGA G-3’
BGLB010031 qPCR	Fwd: 5'-CAT TTT CTA ACC TGA CCC GTT TG-3'
	Rev: 5'-AGT AGC GGT GAT TCT GTT GG-3’
BGLB011379 qPCR	Fwd: 5'-ACG AGA CCT TCT GTG ACA TTC-3'
	Rev: 5'-GTT TTC TTG AAC CCA CTG CC-3’

### Measurement of BgTLR expression in *B*. *glabrata* snails during parasite challenge

Quantitative polymerase chain reaction (qPCR) was used to measure BgTLR expression patterns in schistosome-challenged and control snails. Resistant (BS-90) and susceptible (M-line) snails were exposed individually to ~5 miracidia in 12-well plates. Five snails were collected at selected time points (0, 1, 3, 6, 12 hours and 1, 2, 3, 4, 8, 16, and 35 days post challenge) that represent important milestones in the life cycle of *S*. *mansoni*, and encompass the time from miracidia infection to cercarial shedding in the snail. RNA was extracted from whole snails using a commercial spin column-based kit according to the manufacturer’s instructions (Life Technologies). RNA concentration was determined using a UV/visible spectrophotometer and 1 μg was used for first strand cDNA synthesis. The cDNA was diluted five-fold and 5 μL was used as template in qPCR using primers specific for BgTLR and *B*. *glabrata* β-actin (Table 1), and a dye-based detection system (Quanta Biosciences). Primers were used at final concentrations of 0.6 μM in a reaction volume of 25 μL. The *B*. *glabrata* β-actin gene was used as endogenous control. Snails were confirmed to be *S*. *mansoni* positive or negative using a qPCR assay (S4 Fig) with primers [87] targeting the parasite glyceraldehyde 3-phosphate dehydrogenase (GAPDH) gene. All quantitative PCRs were performed on the ABI 7500 Fast Real-Time PCR system (Applied Biosystems) using the following thermo cycling conditions: initial hold at 95°C for 10 minutes, followed by 40 cycles of 95°C for 15 seconds and 60°C for 1 minute, with data collection every cycle. Specificity for the qPCR amplicons was confirmed by continuous melt curve analysis.

The relative expression of TLR was calculated using the delta-delta cycle threshold method (ΔΔC_t_). In order to generate values for relative expression of TLR transcript normalized to time 0-hour, the cycle threshold values (C_t_) for β-actin were subtracted from TLR C_t_ for the same sample to generate ΔC_t_ values for all samples. Then mean ΔC_t_ for time 0-hour was subtracted from those of the other time points of the same snail strain and treatment to generate ΔΔC_t_ values. Relative quantification (RQ) values were derived from ΔΔC_t_ values using the formula 2^-ΔΔCt^.

### BgTLR knockdown and phenotypic influence on resistance

Juvenile snails (~ 8 mm shell diameter) were injected with a cocktail of 27-mer siRNA oligonucleotides designed to specifically target 4 different regions of the BgTLR transcript. The oligonucleotide sequences were unique to the BgTLR used in this study (S2 Fig) and their specificity was confirmed through qPCR amplifying the three sequences representing transcripts with highest shared nucleotide identity with BgTLR (S5 Fig). The oligonucleotide mix was suspended in Xfect transfection reagent (Clone Tech) in order to enhance delivery and 10–20 μL was injected directly into the snail haemocoel at an approximate final concentration of 6 nM, which was determined by estimating the volume of haemolymph within the snail. Control snails received siRNA oligonucleotides targeting the green fluorescent protein (GFP). Sequences for siRNA oligonucleotides are shown in Table 2.

**Table 2:** siRNA oligonucleotide sequences.

Target^a^	Oligonucleotide sequence
siRNA-BgTLR-1	5ʹ-AGCCAAAUACUAUCGGUCAGUCUCGAC-3ʹ
siRNA-BgTLR-2	5ʹ-GGUCAAAUUGUUAACGCUCAGGUCCAC-3ʹ
siRNA-BgTLR-3	5ʹ-ACGCUGUUUCUGGACAUGUUAGUGGGA-3ʹ
siRNA-BgTLR-4	5ʹ-GUGGACACACAGUUGAAACUUCUUGUC-3ʹ
siRNA-GFP-1	5ʹ-CCAUCAUCUUUGAAGAAGGAACAAUCUUCUUCAAAG-3ʹ
siRNA-GFP-2	5ʹ-AGGUAAUAAUACAGGACCCGGUGAUGGUCCUGUAUU-3ʹ
siRNA-GFP-3	5ʹ-AUGUUGUUACUAAUGUAGCCUUGACCUACAUUAGUA-3ʹ

^a^Targets listed from the 5ʹ to 3ʹ direction of BgTLR coding sequence.

SiRNA-mediated knockdown of BgTLR was demonstrated in both BS-90 and M-line snails without parasite challenge, and BS-90 that have been exposed to *S*. *mansoni*. Five snails were collected for each time point at 3, 12, 24, 48, 72 96 and 120 hours post-injection. RNA extraction, cDNA synthesis and qPCR were performed as described above. BgTLR knockdown was also confirmed at the protein level at the same time points by Western blot using both haemocytes isolated from BS-90 snails and using the *B*. *glabrata* embryonic (Bge) cell line which has haemocyte-like properties [17,88].

Phenotypic influence of BgTLR transcript knockdown was assessed in BS-90 snails. Snails were injected with the BgTLR-specific siRNA oligonucleotide mix and after 48 hours exposed individually to ~5 miracidia. Four weeks later, snails were assessed on a weekly basis, for cercariae shedding. The number of cercariae-producing infections was used as an assessment of the influence of TLR knockdown on the resistance phenotype. BgTLR knockdown snails were compared to BS-90 snails injected with the GFP-specific siRNA oligonucleotides, and to M-line snail controls challenged at the same time.

### Phagocytosis assay

Five BS-90 snails each were injected with siRNA targeting BgTLR or GFP (control) and 96 hours later, haemolymph was extracted from the snails by the head-foot retraction method and immediately mixed with ~1 x 10^6^ 1μM FITC-labelled streptavidin-coated microspheres that were previously incubated with biotinylated *S*. *mansoni* excretory/secretory products and sporocysts [89] following the manufacturers protocols (Bang Laboratories Inc.). Following mixing, the haemolymph was quickly deposited on a microscope slide and allowed to sit in a humidified chamber for 3 hours. The slides were then washed thrice in 1X PBS and examined under a fluorescent microscope. Haemocytes from each snail were counted from a random field of view on the slide, and 30 haemocytes for each snail were assessed for the number of beads within each cell from which percentage phagocytosis (% of cells with one or more beads) and mean number of beads per haemocyte were calculated.

### Immunocytochemistry

To visualize BgTLR in *B*. *glabrata* haemocytes and Bge cells, immunocytochemistry was done using an anti-BgTLR polyclonal antibody raised in rabbits against the extracellular domain of BgTLR (GenScript), and a goat anti-rabbit IgG secondary antibody conjugated to Alexa555 fluorophore (Life Technologies). Snails were bled by head-foot retraction and 100–200 μL of haemolymph was mixed with equal volume of 4% paraformaldehyde prepared in 1X PBS. After 10 minutes of fixation at room temperature, haemocytes were spun at 700 rpm for 5 minutes onto a coverslip and placed in the well of a 6-well plate. Haemocytes were washed with 1 mL of 1X antibody staining buffer (ASB) [0.05% sodium azide, 1% bovine serum albumin (BSA) in 1X phosphate buffered saline (PBS)], then blocked at room temperature for 1 hour in 1% BSA prepared in 1X ASB before staining using the primary antibody (suspended in blocking buffer at 1:200 dilution) at room temperature for 1 hour. Haemocytes were washed three times with 1 mL 1X ASB. Staining in secondary antibody (1:500 dilution) was performed for 1 hour at room temperature and washed as described for the primary antibody. Haemocytes were then mounted in a solution containing DAPI for 5 minutes for the staining of nuclei (GeneTex). Cells from 200 μL of confluent Bge cultures were fixed and treated using the same protocol described for the haemocytes.

Control slides were treated similarly but with primary antibody staining step omitted, or both primary and secondary antibody staining steps omitted. Observation and imaging was done under the LSM710 confocal microscope (Carl Zeiss Microimaging, Germany) at the Cross-Cancer Institute, University of Alberta. Images were processed with the accompanying ZEN 2011 software, version 7.0.0.285 and Photoshop CS5, version 12.0 x64 (Adobe Systems Incorporated, USA).

### Bge cell line

The *Biomphalaria glabrata* embryonic (Bge) cell line is the only existing molluscan cell line. It shares behavioural and molecular attributes with *Biomphalaria* haemocytes such as ability to recognize and phagocytose or encapsulate foreign material including trematode targets [17,88]. Cells were cultured as described by Odoemelam *et al*. [90] and passaged once every 2 weeks by firm tapping or use of cell scrapper to release the cells and reseeding at 1:10 dilution.

### Statistical analysis

To determine significant differences in BgTLR transcript levels, one-way analysis of variance (ANOVA) with Tukey’s post-hoc tests were performed using GraphPad Prism version 6.0f for Mac OS X (GraphPad Software, California USA, www.graphpad.com). Significant differences in the proportions of snails shedding cercariae were determined using the z-test. Statistical significance threshold was set at P ≤ 0.05.

## Supporting Information

S1 Fig Annotated BgTLR coding sequence. The entire open reading frame is shown with alternating lines of nucleotide codons and corresponding amino acid residues. Colour codes represent the start codon (bright green), leucine-rich repeat motifs (pink), transmembrane region (grey), TIR domain (blue) and the stop codon (red).
(TIF)

S2 Fig Nucleotide BLAST of siRNA positions targeted in this study. Sequences representing transcripts with high shared nucleotide identity with BgTLR were retrieved from VectorBase (www.vectorbase.org) and used to create a custom database against which the siRNA sequences were searched. BgTLR is highlighted in grey for each siRNA target results. Percentages in brackets represent the query coverage. Note that BgTLR and BGLB008602-RA are 100% identical in all aspects except that the latter lacks the region between nucleotides 608–1489 (possibly splice variants). Most of the retrieved transcripts have nucleotide conservations of less than 50% to any of the siRNA targets. Shown in the figure is the alignment of 10 top sequences.
(TIF)

S3 Fig Protein and Nucleotide BLAST of BgTLR antibody and qPCR primers respectively. Sequences were analyzed similarly as described for S2 Fig. BgTLR is highlighted in grey for each alignment, displaying up to 10 top sequences. Custom database and BLAST analyses were done using Geneious version 6.1.6 (www.geneious.com) [91].
(TIF)

S4 Fig *Schistosoma mansoni* GAPDH expression. M-line (A) and BS-90 (B) snails were individually exposed to ~5 miracidia (challenged) or left unexposed (not challenged). Five and three snails respectively were collected at indicated time points over the incubation period of the parasite. RNA was extracted from whole snails, converted to cDNA and *S*. *mansoni* GAPDH expression was measured by quantitative PCR. All snails having a cycle threshold (C_t_) value above zero were considered infected. RNA extracted from miracidia was used as a positive control template.
(TIF)

S5 Fig siRNA-mediated knockdown of BgTLR does not affect other transcripts with a high shared nucleotide identity with BgTLR. Quantitative PCR was performed targeting 3 transcripts that appear to be the most similar to BgTLR: BGLB008602 (A), BGLB010031 (B) and BGLB011379 (C) using cDNA generated from BS-90 BgTLR and GFP siRNA knockdown samples as templates. BgTLR siRNA did not have knockdown effect on these TLRs except on the putative splice variant (BGLB008602) which displayed a pattern similar to BgTLR knockdown.
(TIF)

S6 Fig BgTLR is not expressed on all haemocytes. Bright field view (A) of two adjacent haemocytes labelled with the nuclear stain DAPI (B) and anti-BgTLR primary antibody (C). The merged view (D) shows that BgTLR protein was only expressed on the haemocyte on the right of the panel. Scale bars represent 20 μM.
(TIF)

S7 Fig BgTLR detection antibody is specific to its cognate peptide. Protein extracts from BS-90 haemocytes were ran on duplicate SDS-PAGE gels, then transferred to nitrocellulose membranes and probed with BgTLR antibody used in this study without pre-incubation with its cognate peptide [–], pre-incubation with the peptide at 2:1 (peptide:antibody) molar ratio [3(2:1)], equal molar ratio [3(1:1)] or with an alternative BgTLR antibody targeting a different peptide [1(2:1)]. *B*. *glabrata* actin served as protein loading control. M = molecular marker.
(TIF)

S1 Table Membrane-associated proteins displaying differential expression in haemocytes of BS-90 *B*. *glabrata*. (DOCX)

S1 Video Video of images acquired from multiple planes (z-stacks) supporting the description of BgTLR as plasma membrane-associated. Samples were processed as described in materials and methods and 0.5 μM image slices were acquired under the LSM710 confocal microscope and processed into a video with the accompanying ZEN 2011 software, version 7.0.0.285.
(AVI)
