# Supplementary figures and images for: A Novel Toll-Like Receptor (TLR) Influences Compatibility between the Gastropod Biomphalaria glabrata, and the Digenean Trematode Schistosoma mansoni
Source: PLoS Pathog. 2016 Mar 25;12(3):e1005513. doi: 10.1371/journal.ppat.1005513 (PMC4807771; doi:10.1371/journal.ppat.1005513)

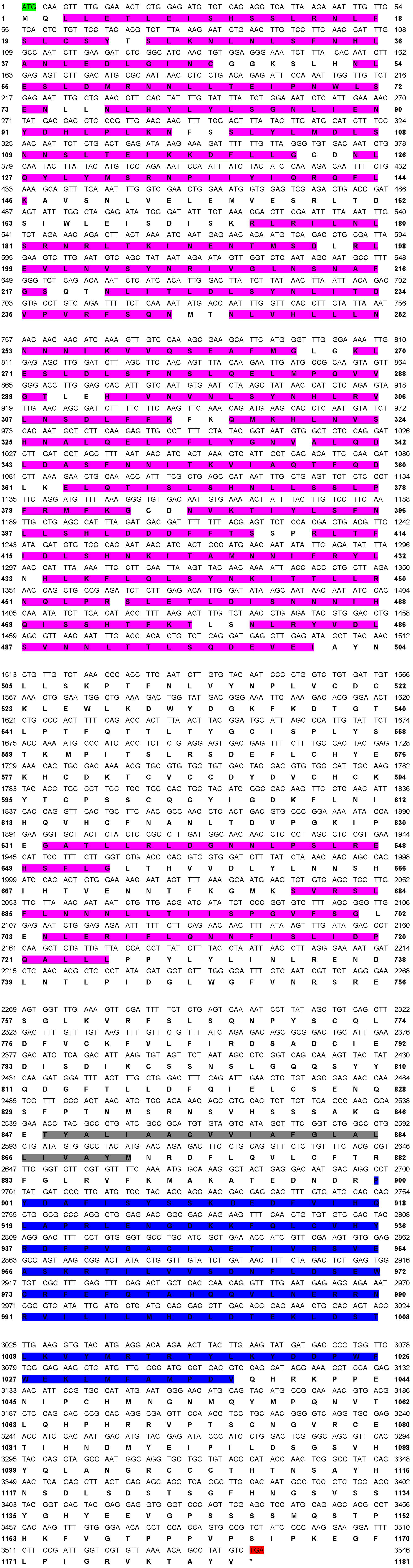

Supplement: S1 Fig — The entire open reading frame is shown with alternating lines of nucleotide codons and corresponding amino acid residues. Colour codes represent the start codon (bright green), leucine-rich repeat motifs (pink), transmembrane region (grey), TIR domain (blue) and the stop codon (red). (TIF) [file ppat.1005513.s001.tif]

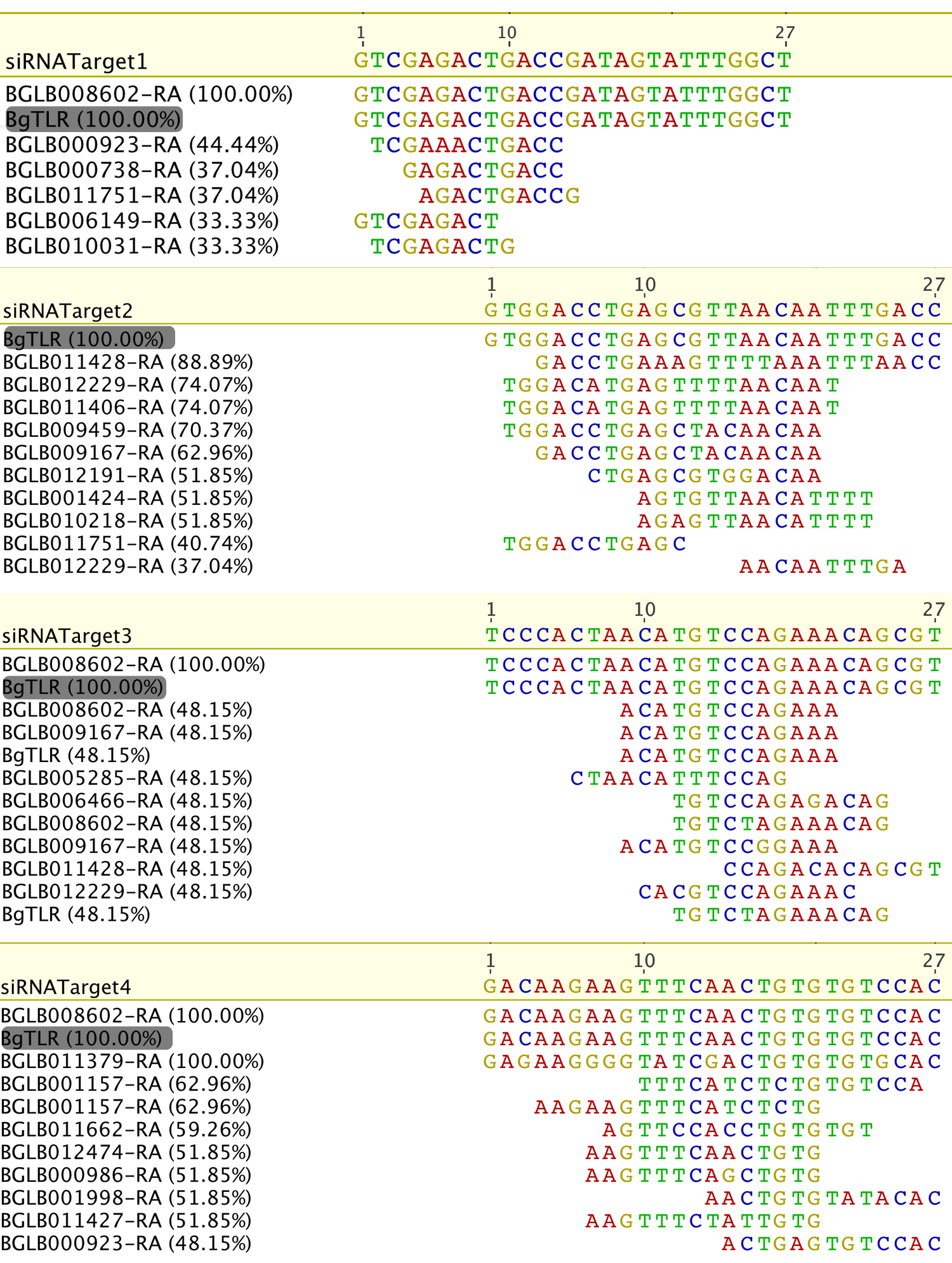

Supplement: S2 Fig — Sequences representing transcripts with high shared nucleotide identity with BgTLR were retrieved from VectorBase (www.vectorbase.org) and used to create a custom database against which the siRNA sequences were searched. BgTLR is highlighted in grey for each siRNA target results. Percentages in brackets represent the query coverage. Note that BgTLR and BGLB008602-RA are 100% identical in all aspects except that the latter lacks the region between nucleotides 608–1489 (possibly splice variants). Most of the retrieved transcripts have nucleotide conservations of less than 50% to any of the siRNA targets. Shown in the figure is the alignment of 10 top sequences. (TIF) [file ppat.1005513.s002.tif]

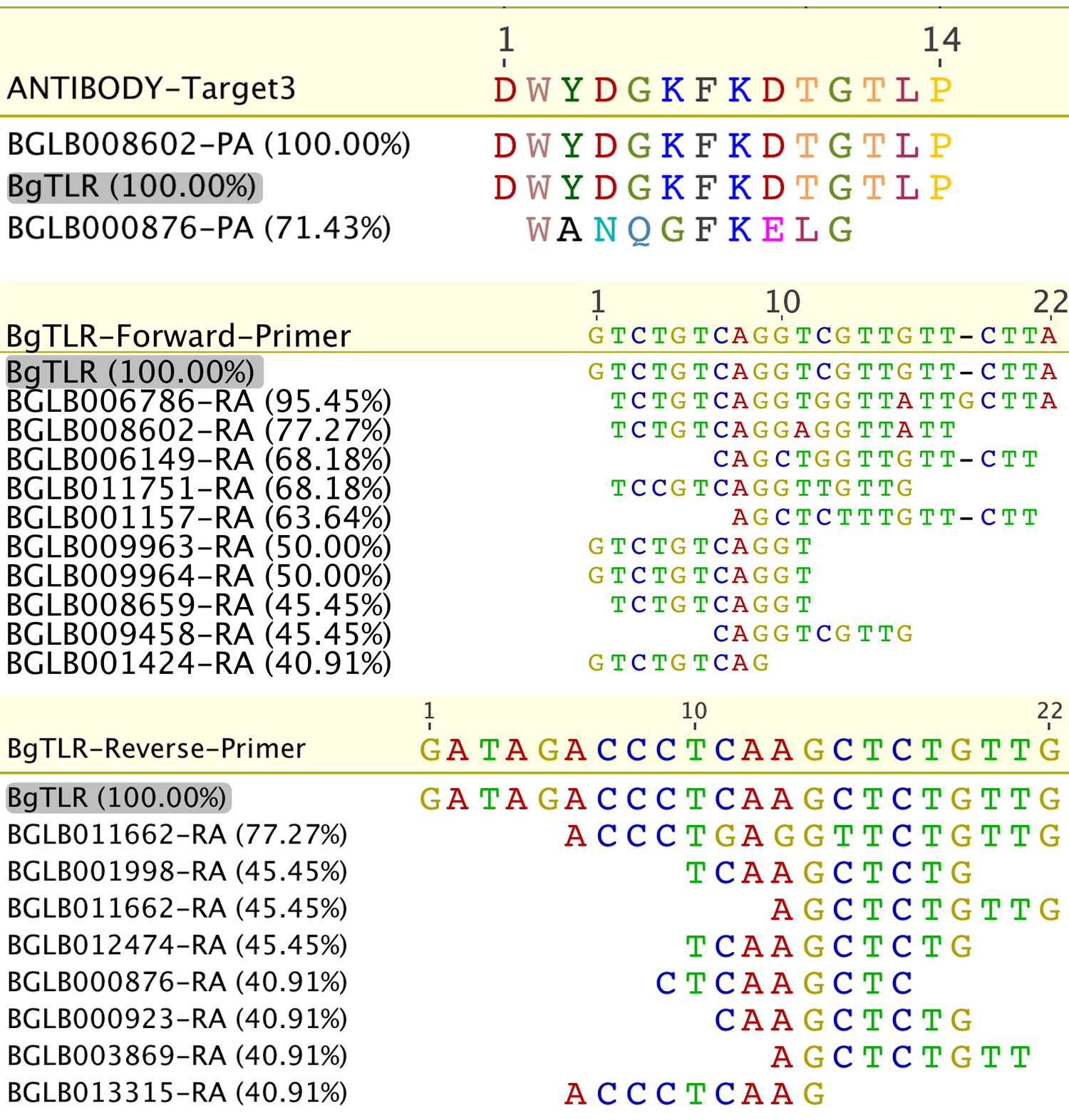

Supplement: S3 Fig — Sequences were analyzed similarly as described for S2 Fig. BgTLR is highlighted in grey for each alignment, displaying up to 10 top sequences. Custom database and BLAST analyses were done using Geneious version 6.1.6 (www.geneious.com) [91]. (TIF) [file ppat.1005513.s003.tif]

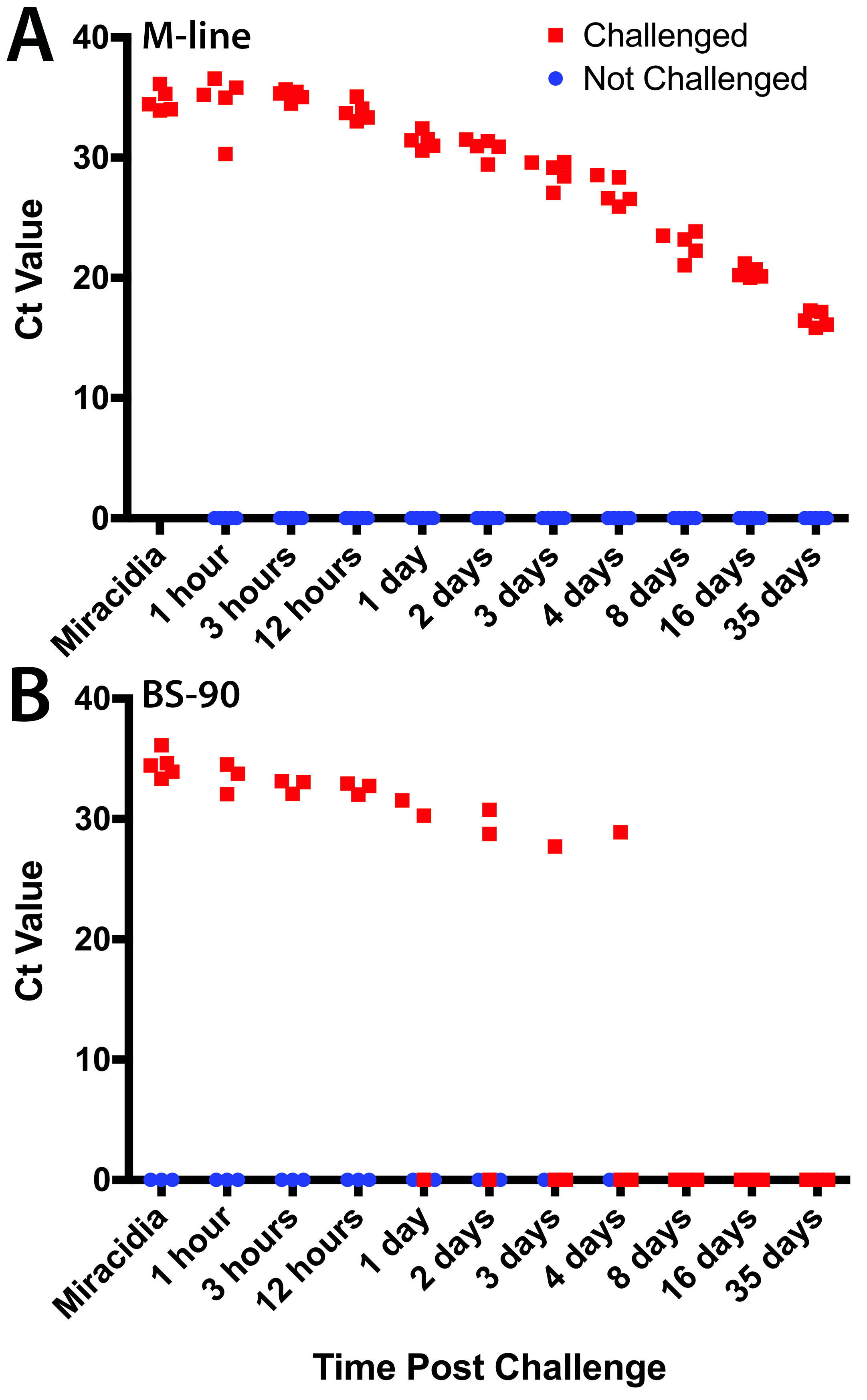

Supplement: S4 Fig — M-line (A) and BS-90 (B) snails were individually exposed to ~5 miracidia (challenged) or left unexposed (not challenged). Five and three snails respectively were collected at indicated time points over the incubation period of the parasite. RNA was extracted from whole snails, converted to cDNA and S. mansoni GAPDH expression was measured by quantitative PCR. All snails having a cycle threshold (Ct) value above zero were considered infected. RNA extracted from miracidia was used as a positive control template. (TIF) [file ppat.1005513.s004.tif]

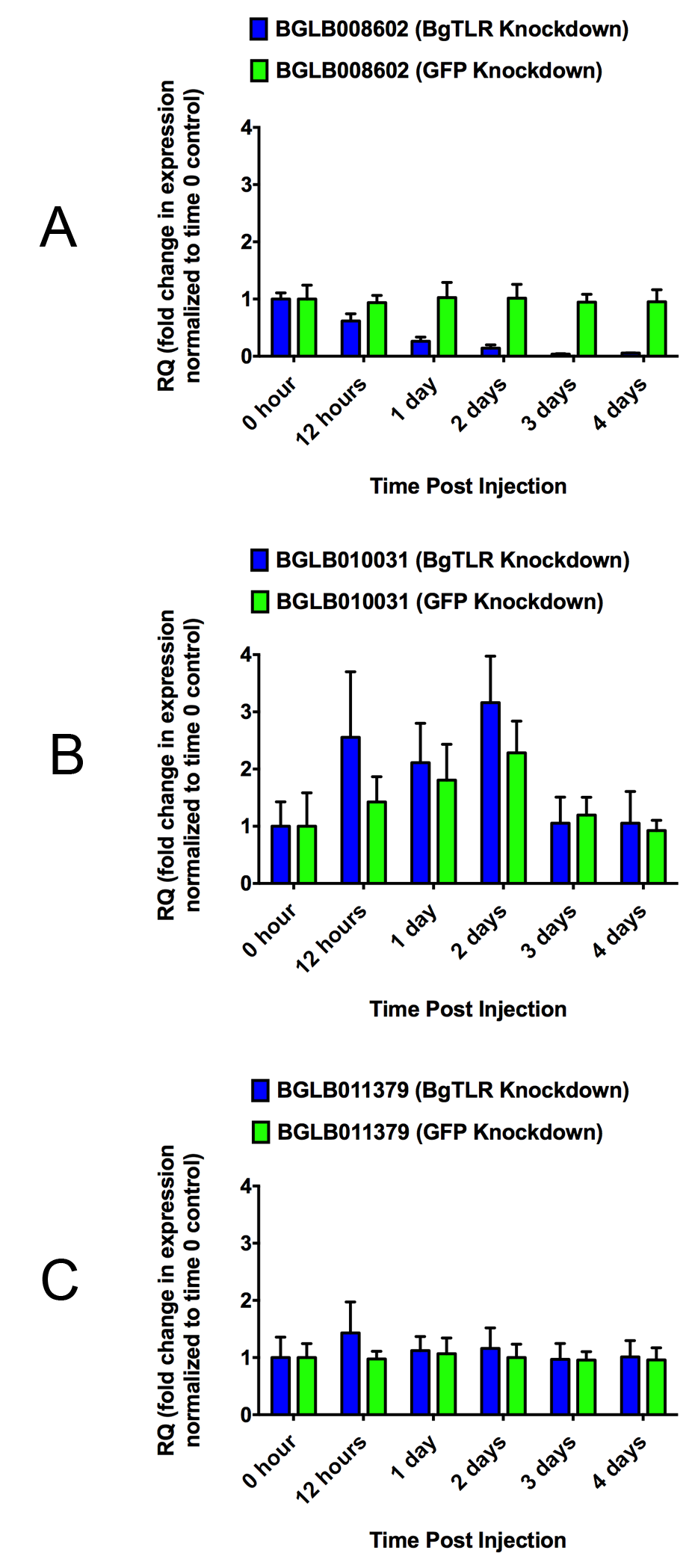

Supplement: S5 Fig — Quantitative PCR was performed targeting 3 transcripts that appear to be the most similar to BgTLR: BGLB008602 (A), BGLB010031 (B) and BGLB011379 (C) using cDNA generated from BS-90 BgTLR and GFP siRNA knockdown samples as templates. BgTLR siRNA did not have knockdown effect on these TLRs except on the putative splice variant (BGLB008602) which displayed a pattern similar to BgTLR knockdown. (TIF) [file ppat.1005513.s005.tif]

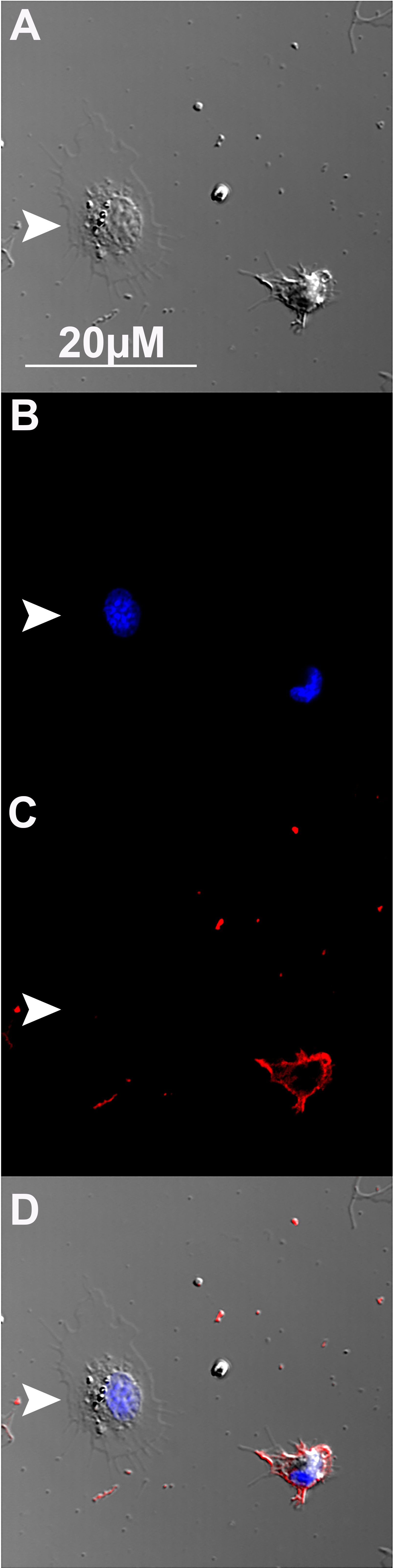

Supplement: S6 Fig — Bright field view (A) of two adjacent haemocytes labelled with the nuclear stain DAPI (B) and anti-BgTLR primary antibody (C). The merged view (D) shows that BgTLR protein was only expressed on the haemocyte on the right of the panel. Scale bars represent 20 μM. (TIF) [file ppat.1005513.s006.tif]

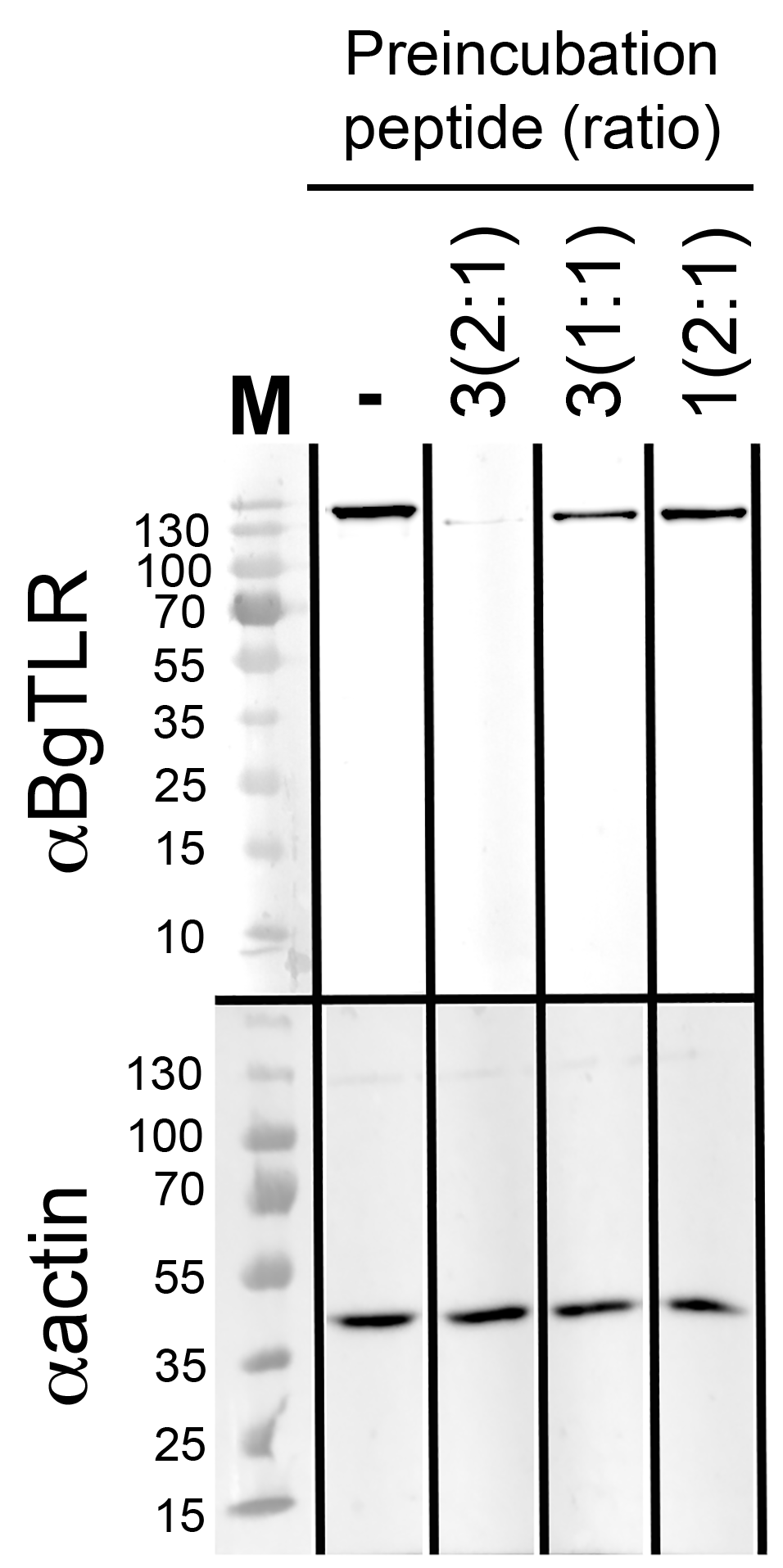

Supplement: S7 Fig — Protein extracts from BS-90 haemocytes were ran on duplicate SDS-PAGE gels, then transferred to nitrocellulose membranes and probed with BgTLR antibody used in this study without pre-incubation with its cognate peptide [–], pre-incubation with the peptide at 2:1 (peptide:antibody) molar ratio [3(2:1)], equal molar ratio [3(1:1)] or with an alternative BgTLR antibody targeting a different peptide [1(2:1)]. B. glabrata actin served as protein loading control. M = molecular marker. (TIF) [file ppat.1005513.s007.tif]
