# Supplementary material for: A Novel Toll-Like Receptor (TLR) Influences Compatibility between the Gastropod Biomphalaria glabrata, and the Digenean Trematode Schistosoma mansoni
Source: PLoS Pathog. 2016 Mar 25;12(3):e1005513. doi: 10.1371/journal.ppat.1005513 (PMC4807771; doi:10.1371/journal.ppat.1005513)
Supplement: S1 Table — (DOCX) [file ppat.1005513.s008.docx]

| **Protein with highest amino acid identity identified in Genbank** | **Accession #** | **Protein coverage (%)** | **Closest *B. glabrata* match** |
| --- | --- | --- | --- |
| BgTLR | AGB93809 | 24 |  |
| Superoxide dismutase Cu/Zn | AAR98628 | 38 |  |
| Glutathione S-transferase | XP_013064657 | 8 |  |
| Histone H3 | XP_013086290 | 16 |  |
| Hypothetical protein |  | 12 | EW997021 |
| Hypothetical protein |  | 8 | CV548474 |
| alpha-2 Actin | AF329436_1 | 33 |  |
| Myosin II | AF497246_1 | 26 |  |
| Dermatopontin 2 | AAZ80785 | 37 |  |
| Matrilin | AAZ80784 | 28 |  |
| Similar to transmembrane protease (Elastase 2-like) |  | 16 | EW996827 |
| Elongation factor 1 alpha | AHH81789 | 24 |  |
| Fibropellin-1 like (*Xenopus tropicalis*) | XP_012810771 | 10 | EW996942 |
| Unknown |  | 9 | EW997199 |
| Unknown |  | 7 | EW997226 |
| PREDICTED: protein tyrosine phosphatase domain-containing protein 1-like | XP_013081291 | 19 |  |

**S1 Table. Membrane-associated proteins displaying differential expression in haemocytes of BS-90 *B. glabrata.***
